# A Practical Monitoring System for the Structural Safety of Mega-Trusses Using Wireless Vibrating Wire Strain Gauges

**DOI:** 10.3390/s131217346

**Published:** 2013-12-16

**Authors:** Hyo Seon Park, Hwan Young Lee, Se Woon Choi, Yousok Kim

**Affiliations:** 1 Department of Architectural Engineering, Yonsei University, 134 Shinchon-dong, Seoul 110-732, Korea; E-Mails: hspark@yonsei.ac.kr (H.S.P.); ieehyoung@yonsei.ac.kr (H.Y.L.); 2 Center for Structural Health Care Technology in Buildings, Yonsei University, 134 Shinchon-dong, Seoul 110-732, Korea; E-Mail: watercloud@yonsei.ac.kr

**Keywords:** vibrating wire strain gauge, structural health monitoring, wireless network, mega-truss, temperature stress, long-term monitoring, irregular building

## Abstract

Sensor technologies have been actively employed in structural health monitoring (SHM) to evaluate structural safety. To provide stable and real-time monitoring, a practical wireless sensor network system (WSNS) based on vibrating wire strain gauges (VWSGs) is proposed and applied to a building under construction. In this WSNS, the data measured from each VWSG are transmitted to the sensor node via a signal line and then transmitted to the master node through a short-range wireless communication module (operating on the Industrial, Scientific, and Medical (ISM) band). The master node also employs a long-range wireless communication module (Code Division Multiple Access—CDMA) to transmit the received data from the sensor node to a server located in a remote area, which enables a manager to examine the measured data in real time without any time or location restrictions. In this study, a total of 48 VWSGs, 14 sensor nodes, and seven master nodes were implemented to measure long-term strain variations of mega-trusses in an irregular large-scale building under construction. Based on strain data collected over a 16-month period, a quantitative evaluation of the construction process was performed to determine the aspects that exhibit the greatest influence on member behavior and to conduct a comparison with numerical simulation results. The effect of temperature stress on the structural elements was also analyzed. From these observations, the feasibility of a long-term WSNS based on VWSGs to evaluate the structural safety of an irregular building under construction was confirmed.

## 1. Introduction

Visual inspection has traditionally played a critical role in quality management of the construction process and damage detection in structures subjected to various loadings [1,2]. However, because structures have become increasingly complex (e.g., high-rise and irregular designs), visual inspections are becoming increasingly time consuming and labor intensive and suffer from expensive and subjective evaluations; these aspects represent critical problems in the application of this method to real structures. Because visual inspection is restricted to post-event assessments, immediate damage detection and safety evaluations of structures are nearly impossible.

For these reasons, structural health monitoring (SHM) based on sensor technology has received considerable attention and has successfully replaced traditional visual inspection [3–6]. The SHM of buildings, which is based on a wired sensor network, was initially conducted for simple civil structures. The development of monitoring systems enabled a real-time response evaluation of a structure. However, the high installation cost of the cable that connects the sensor to the server and maintenance and management challenges remain unresolved issues.

For these reasons, Straser [7] attempted to solve the problems of existing wire-based monitoring systems by proving the effectiveness of the wireless sensor network (WSN) in an actual building. The WSN system (WSNS) significantly decreased the installation cost of a wired sensor network, and thus, the high-nodal density of sensor networsks was realized, which enabled local damage detection. Its applications and state-of-the-art design are well documented [7–12]. Different types of sensors have been employed in SHM to measure the responses of structures, such as acceleration [13–16], displacement [17–21], and strain [22–26].

In special circumstances, strain-type sensors are adopted to directly measure the strain of a structural element. The stress distribution estimated from a strain measurement can be utilized in a safety assessment of an element by comparing it with the yield stress of materials or the design strength of structural members. Several types of strain gauges are used to monitor structural responses, including electrical strain gauges (ESGs), fiber optic sensors (FOSs, [27–31]), and vibrating wire strain gauges (VWSGs, [32–34]). Among these strain-type sensors, FOSs and VWSGs, which are immune to electromagnetic interference and provide superior endurance, are actively studied and employed in SHM. Despite the outstanding potential of FOSs in health monitoring applications [27], FOSs are extremely fragile, which contributes to a high rate of installation failure in real structures. In addition, both the measurement system and sensors are relatively expensive compared with VWSGs. Other advantages of VWSGs include the ease and low cost of installation and the use of a smaller amount of data logger bandwidth due to the simple measurement principle of VWSGs. They are appropriate for long-term monitoring because vibrating wires exhibit minimal deterioration over time.

The accuracy and reliability of VWSGs as sensors for measuring strain have been verified from numerous laboratory experiments [35–38]. However, few studies have focused on its application in actual building construction, for which numerous obstacles hinder stable measurements. Although some researchers have conducted real-time monitoring using VWSGs in high-rise buildings during construction [39], the development of WSNS for buildings under construction remains a unique and challenging task.

As a practical monitoring technique, a WSNS based on VWSGs is proposed to obtain reliable data that can enhance structural safety and construction precision through long-term real-time monitoring. The proposed WSNS realized the functions required by wireless network systems through the application of a power-saving wireless VWSG sensor node, which offers three major built-in functions: data collection, data processing, and data transmission. With the exception of the signal cable that connects the sensor to the sensor node, the proposed system enables the entire network to be wireless. Therefore, an automatic system in which the manager can examine data collected from VWSGs in real time was realized.

The structural system of the subject building consists of two mega-trusses, which were installed above four mega-columns to form a three-dimensional irregular shape on a maximum scale. These mega-structures were designed to support the floor and roof of a large space. The mega-truss, whose length extends 35 m, is the cantilever structure and one of the main members that ultimately supports the other members; thus, precise construction is required during construction.

Because stress changes in the main structural elements are anticipated during subsequent phases of construction, a total of 48 VWSGs, 14 sensor nodes, and seven master nodes were implemented to monitor the strain variation in the mega-truss of the irregular large-scale building under construction. Based on strain data collected over a long-term monitoring for 16 months, a quantitative evaluation of the construction process was performed to determine the aspects that exhibit the greatest influence on member behavior and their effects. The numerical model was also verified by comparing the measurement results with the simulated results obtained from the preliminary analysis. In addition, variations in temperature during construction were obtained from temperature sensors, which were installed on major elements and used to investigate the effects of temperature stress on the structural elements. The feasibility of the proposed long-term monitoring system, which is based on wireless VWSGs, to evaluate the structural safety of an irregular building under construction was investigated.

## 2. WSNS Using VWSGs

**Figure 1.**
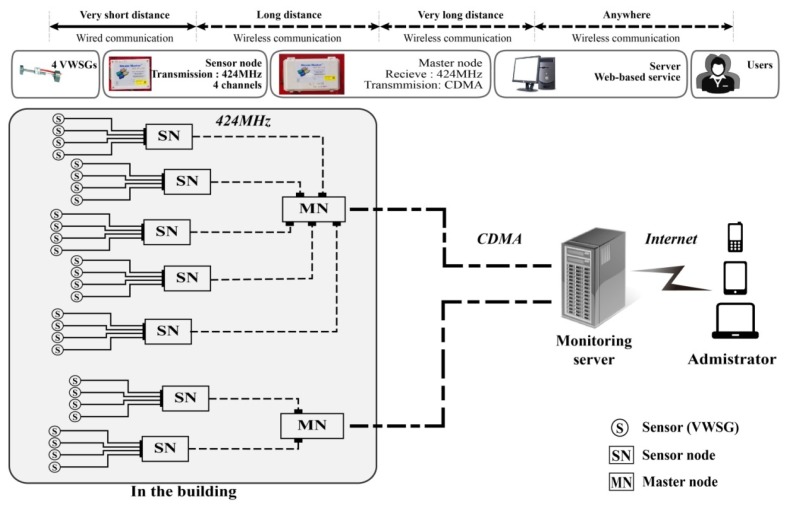
WSNS.

A WSNS based on VWSGs consists of two nodes: the sensor node, which processes and transmits the raw data obtained from the VWSGs to the other network equipment (master node), and the master node, which receives all data from the sensor node and transmits the data to the monitoring server. As illustrated in Figure 1, the entire network of the WSNS in this study was wirelessly automated for the convenience of measurement and maintenance and was constructed so that the user can observe data in real time.

### 2.1. VWSG Sensor

**Figure 2.**
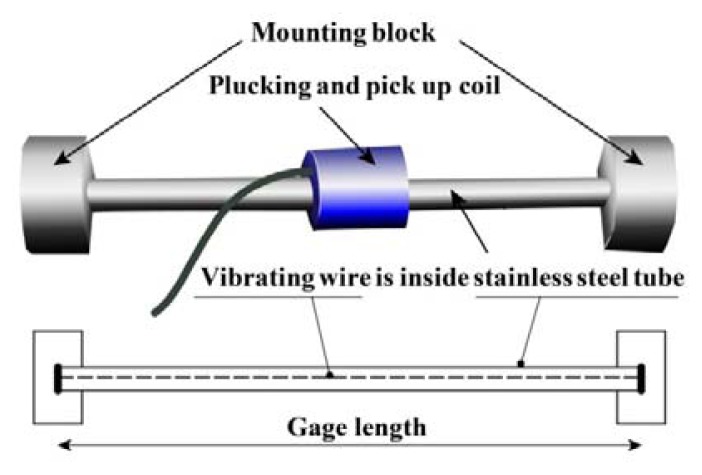
VWSG.

The VWSG has a long transmission length compared with an electrical resistance strain gauge. It also has a lower exterior electromagnetic effect, is less affected by vibration and impact force, and enables semi-permanent measurements. Longitudinal deformation of the wire can occur due to tension and compression, and the natural frequency of the VWSG can vary accordingly. This output frequency measures the average strain for the length of the vibrating wire gauge connected between the mounting blocks (Figure 2). The strain ε can be simply calculated with the change in frequency as:

(1) $$\in \;=k\;\left(f_{2}^{2}-f_{1}^{2}\right)$$

where *k* is the gauge factor determined by the properties of the vibrating wire and length and *f*_1_ and *f*_2_ denote the natural frequency prior to and after a change, respectively.

### 2.2. Wireless Sensor/Master Nodes

**Figure 3.**
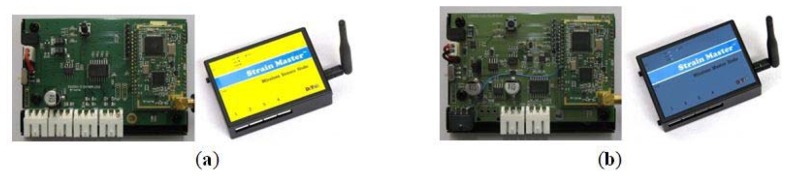
Wireless nodes. (**a**) Sensor node; (**b**) Master node.

The VWSGs were grouped (a maximum of four) and connected through a signal cable to the sensor node located near the sensors. Each sensor node with a four-channel sensor interface can simultaneously receive and process data from four VWSGs (Figure 3a, [34]). The raw data from the VWSGs are processed by the data processor built into the sensor node. The processed data are transmitted to the master node through a short-range (Industrial, Scientific, and Medical band—ISM band) wireless communication module. Further information about the wireless sensor node is available in [34].

The master node shown in Figure 3b [40,41] receives processed data from the sensor nodes through short-range wireless communication (ISM band) and transmits these data to the monitoring server through long-range (Code Division Multiple Access—CDMA, [42]) communication. Four sensor nodes can be connected to each master node with a four-port interface, which can simultaneously receive data from 16 VWSGs via sensor nodes.

The communication between the sensor node and master node uses an ultra-high-frequency ISM band of 424 MHz. Compared with microwaves, there is less influence from interference and adequate diffraction of obstacles, thus, the ISM band is considered a suitable communication method for a construction site. A valid communication distance for short-range wireless communication with a sensor node is within 400 m with line of sight (LOS) and within 100 m with non-line of sight. The operating temperature range is approximately −30 to 85 °C.

The wireless communication method of CDMA between the master node and server involves spread-spectrum communication, thus, there is practically no loss of data during transmission and the method is suitable for long-term monitoring due to its low power consumption. Regardless of the communication distance, numerous managers can access the data at preferred times with a personal computer (PC), notebook, tablet PC, mobile phone, or other wireless communication in locations where Internet access is available.

Regarding power consumption, which has been a critical issue for WSNS, this research employed a power-saving circuit in sensor and master nodes, which activated the equipment to operating mode only when the network measurement equipment was in the process of measuring or transmitting. When not under these conditions, the system converted to sleep mode to minimize energy consumption. To apply such low-power technology, the measurement and transmission of all network equipment was synchronized. Only 170 μA of power was consumed during sleep mode, which enabled long-term use in locations that lacked a continuous supply of electricity.

## 3. Application of the WSNS

### 3.1. Target Structure: Mega-Truss in the D-Building

**Figure 4.**
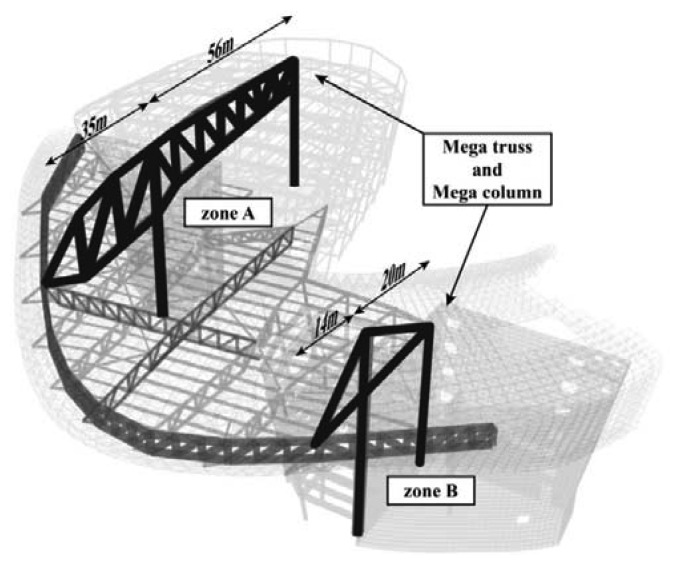
Structural frames.

The subject building for the application of the WSNS based on VWSGs is the D-Building, which is located in Seoul, South Korea. The main structural frame, which is a reinforced-concrete (RC) and steel structure, consists of three underground floors and four stories, as shown in Figure 4. The steel structure of the mega-truss and mega-column comprise the main frames that form the three-dimensional curve of the streamline design. Zones A and B each contain one mega-truss, which is a large member with a depth of approximately 5–14 m. In zone A, two mega columns support the mega-truss, whose total length is 91 m; the length of the cantilever is 35 m. In zone B, two mega-columns support the mega-truss, whose total length is 34 m; the length of the cantilever is 14 m.

The main gravity load that is directly conveyed to the mega-truss includes the floor load of the fourth floor and the finishing load of the roof level. The steel material consisted of SM570 with a yield strain and yield strength of 2,136 με and 355 MPa, respectively.

### 3.2. WSNS in the D-Building

**Figure 5.**
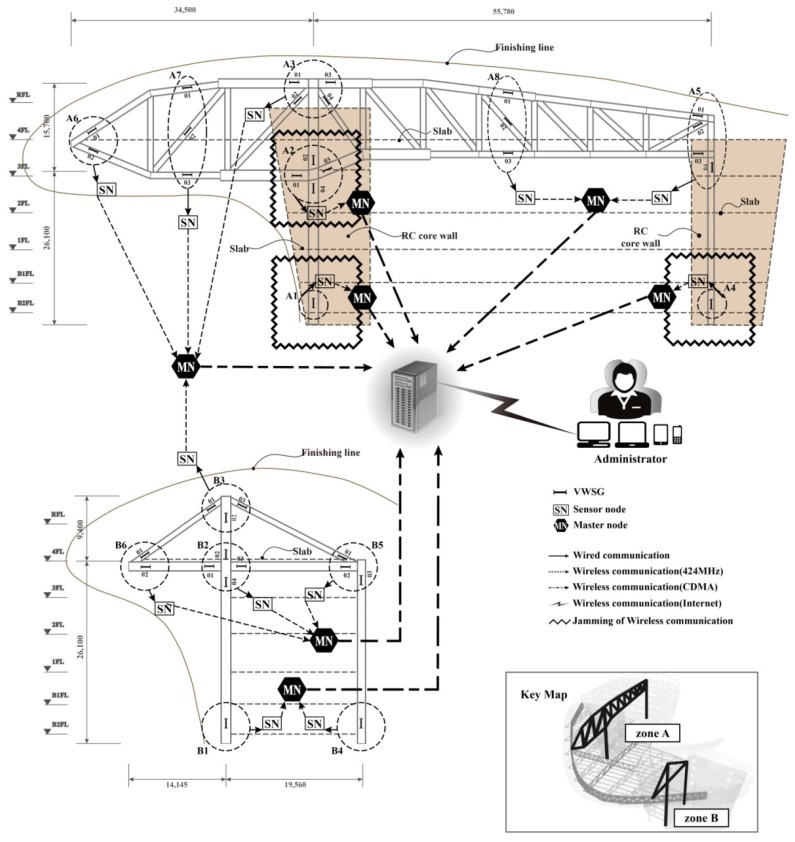
WSNS for the D-Building.

**Figure 6.**
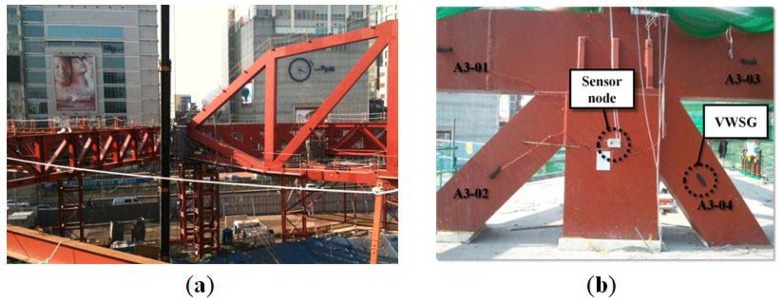
Application site during construction. (**a**) Mega-truss (A6 and A7 areas) during construction; (**b**) VWSG and sensor node of zone A3 in the D-Building.

A total of 48 VWSGs, 14 sensor nodes, and seven master nodes were applied to the mega-truss, on which long term monitoring for 16 months was conducted. As shown in Figure 5, the mega-truss and mega-column were divided into monitoring areas A1–A8 and B1–B6. Figure 6a shows A6 and A7 areas of the mega-truss during construction. The areas were divided based on adjacent sensors, which were connected to one sensor node through signal lines to narrow the wire range (signal line), as shown in Figure 6b. This process heightened the applicability of on-site measurements in the building during construction.

The raw data measured by the VWSGs are transmitted to the sensor node and pass through the data processing system built into the sensor node. The communication jamming ( 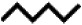), which is shown in Figure 5, is caused by physical factors, such as the thick RC wall and slab without openings. Therefore, a separate master node was installed. Because other truss areas are exposed to the exterior, the master nodes were arranged based on the communication distance from the sensor node to other obstacles. The master node transmits all processed strain data to the monitoring server through the long-range wireless communication method of CDMA.

## 4. Analysis of Strain Data from the VWSG during Construction

### 4.1. Relationship between Construction Events and the Observed Strain Data

**Figure 7.**
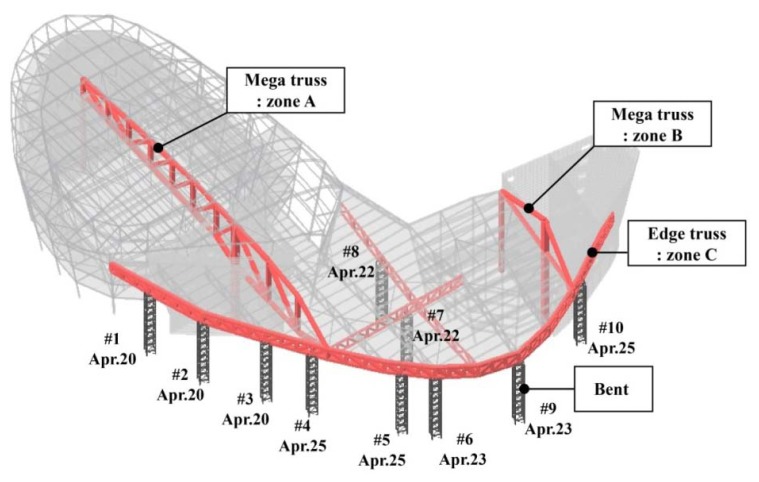
Removal schedule of temporary bents.

The variations in the measured strain data according to the various events of the construction schedule are evaluated in this section. The main construction events are listed in Table 1. The bents were installed to secure stability during the construction of the mega-truss, edge truss, and floor truss. As shown in Figure 7, there are a total of 10 bents on the mega-truss: two bents support the frame at the end of the cantilever of the mega-truss (#4 and #10), and a total of eight bents exist on the edge truss and floor truss. When the erection of the framework was nearly complete, the temporary bents were removed in the sequential order shown in Figure 7. Subsequently, the self-weight of the structure, which had been supported by the bents, was relocated to the mega-truss. Therefore, substantial vertical displacements and stress variations were expected to occur in the cantilever-type mega-truss and edge truss. Bent removal (Event 4 in Table 1) was the most significant event for the stability and precise construction of the building during the monitoring period in this study.

**Figure 8.**
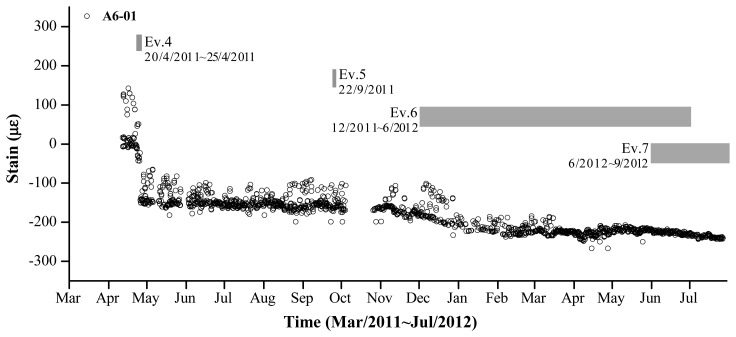
Construction events and strain of A6-01.

Figure 8 displays the variations in strain data measured by the VWSG that was installed in zone A6-01 according to the construction schedule (events 4–7). A substantial change occurred in the measured value from April 25, 2011, when all bents were removed. After event 4, minimal strain variations were observed until event 5 was initiated. Conversely, during event 5–7, the self-weight of the concrete, space-frame, and exterior panels on the fourth floor were applied, causing strain variations in the mega-trusses. However, their effect on strain variations was insignificant compared with the effect of the bent removal on strain variation.

**Figure 9.**
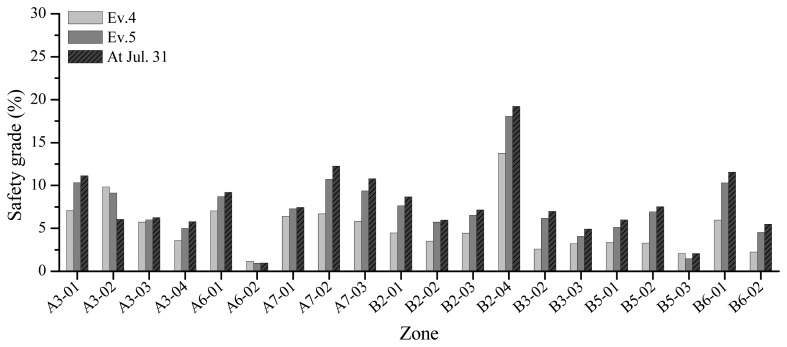
Structural safety during construction events.

Figure 9 illustrates the safety grades according to the construction schedule. The safety grade signifies the ratio of the measured strain of the member to the yield strain of the member, which comprise the cumulative results until each event was finished. Although the variation in the measured data is in accordance with the construction events, the safety grade increases to the maximum value of approximately 15% at B2-04 after complete removal of the bents.

### 4.2. Comparison of the Predicted and Measured Strain Responses

This section presents a comparison of the measured strain data for the mega-truss during the removal of the bents with the predicted results obtained from the preliminary analysis. The analysis program MIDAS/GEN Ver. 800 [43] was employed for the comparison. The mega-truss has a full-strength butt welding (FSBW) of approximately 2 m from the working point (WP); thus, a moment connection was applied to the modeling to reflect its structural integrity. Regarding the modeling, the mesh was divided into plate elements for the irregularity of the walls and the accuracy of the model. To correctly reflect the rigidity of the core and fourth slab, these elements were modeled with plate elements that were similar to the wall. The temporary bents were modeled according to the actual building data such that the vertical load was conveyed on the point where the bent and main structure connected. The effect of bent removal in the analysis model was considered by removing the points of the mega-truss and edge truss, which were supported by the bent, and modifying them to free ends. The main evaluating period for the measured data began on April 20, 2011, when bent removal commenced, and ended on April 25, 2011, when all temporary bents had been removed.

**Figure 10.**
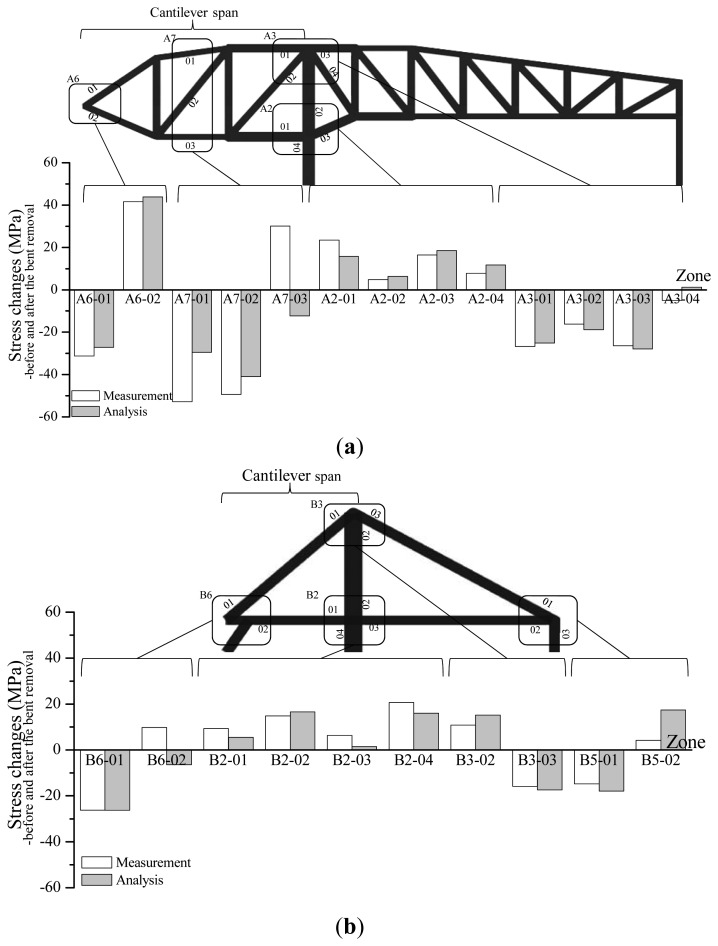
Stress changes before and after bent removal. (**a**) Zone A; (**b**) Zone B.

A comparison of the measured data for all members with the predicted values reveals the variations in stress prior to removal of the bents and after the complete removal of the last bent, as shown in Figure 10. A3-01 and A2-01 are horizontal members fixed to the mega-column (Figure 5). After the removal of bent #4 in Figure 7, the horizontal members became the fixed ends of the cantilever structure. Due to the deflection of the free end (point of bent removal), which is caused by self-weight, the tensile stress was generated in the upper member of A3-01 (−26 MPa) and compressive stress (+23.4 MPa) occurred in the lower member of A2-01. The largest changes in stress occurred at A6 and A7, in which a significant change in support occurred. The stress variation in zone B is smaller than that in zone A because the former has a smaller cantilever length (14.1 m *versus* 34.5 m).

These results indicate that the preliminary analysis results accurately reflect the tendency of stress variation in the mega-truss members during bent removal, although a slight discrepancy exists between the calculated and measured strain data in quantitative terms. A more accurate analysis model may be constructed through an in-depth review of the measured results.

### 4.3. Thermal Stress Effect on Structural Responses

**Figure 11.**
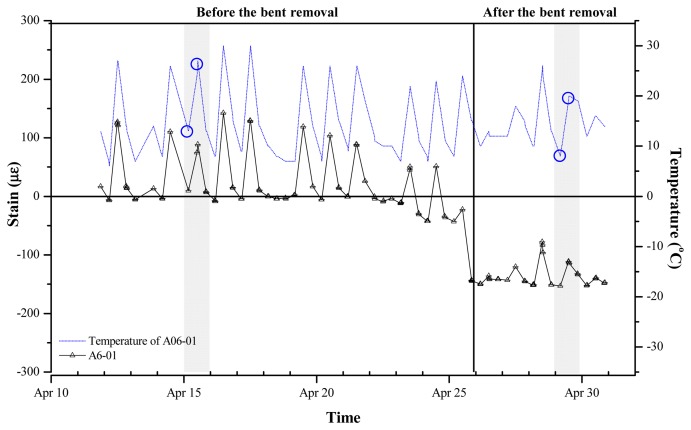
Relationship between temperature and strain (A6-01).

Figure 11 shows the variations in strain rates (solid line) and temperature (dotted line) which is the result of data collection three times a day (once every 8 h). Although the measurements were collected on a single day, there is a substantial disparity in their values. That is, the strain values are fluctuating with variation of temperature. It may be attributable to the spring temperatures in South Korea, which exhibit an expansive daily temperature range. The daily maximum temperature at the D-Building construction site was 20 °C.

To extensively analyze the relationship of strain regarding temperature variation in this area, the temperature stress change for each member can be expressed as:

(2) $$\Delta \sigma_{T}=R\cdot E\cdot \Delta T$$

where Δσ*_T_* is the stress change of the members according to the temperature change and *R* is the coefficient of thermal expansion of the member considering the confining effect of the frame. If the steel member is not confined, there is a linear expansion of 12 × 10^−6^/°C for every temperature increase of 1 °C. *E* represents the Young's Modulus of the mega-truss, and Δ*T* is the temperature difference. When the member is within the elastic range, the following formula is valid according to Hook's Law:

(3) Δσ=E⋅Δε

Therefore, when there is no load other than the temperature load, the stresses from Equations (2) and (3) are equal. For a member in the elastic range, *R* can be expressed as follows:

(4) $$R=\frac{\Delta \varepsilon }{\Delta T}$$

**Figure 12.**
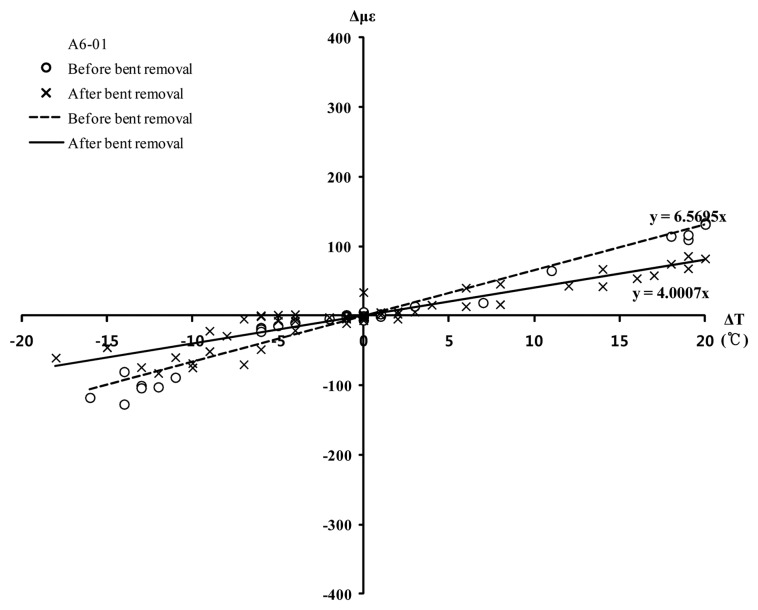
Change in thermal coefficient (*R*) of A6-01.

Figure 12 shows the relationship of strain versus temperature change for the A6-01 sensor. Formula (4) represents the gradient in Figure 12. When the gradient is negative, tensile stress occurs in the member; when the gradient is positive, compressive stress occurs in the member.

The change in the R value before and after bent removal was examined through linear approximation. The results indicate that the coefficient R was approximately 6.6 × 10^−6^ prior to bent removal but decreased to approximately 4.0 × 10^−6^ after bent removal. Because the R value is positive before and after bent removal, additional compression is applied with increasing temperature. The absolute value |R| is smaller after bent removal; thus, the resulting change in stress due to temperature is smaller. For example, when there is a 10 °C increase in temperature, an additional compressive stress of 13.5 MPa is produced by the temperature prior to bent removal; however, after bent removal, an additional compressive stress of 8.2 MPa is produced. This difference may be due to the varying degrees of restraint from the change in supports, which resulted from bent removal. Considering that the maximum strain, which occurred during bent removal, was approximately 200 με, the strain caused by the variations in temperature is significant compared with the strain caused by bent removal.

## 5. Conclusions

This research developed a long-term (16-month) real-time WSNS using VWSGs, which were applied to an irregular building under construction. The data measured by the sensors (VWSGs) are transmitted to the sensor node, and the raw data are processed by the built-in ADC and data processor. The data processed in the sensor nodes are transmitted to the master node through a short-range wireless communication module (ISM), and the master node sends the received data to the monitoring server through long-range wireless communication (CDMA). Thus, the WSNS enables the user to review the measured data in real time. The structural response measurements that were conducted for the large-scale structural members (mega-truss), which support the large space of the structure, are described below.

By examining the stress variations in the mega-truss during load changes for each construction event, it was confirmed that the bent removal process exhibited the largest influence on the structural member. Bent removal, which was performed after the construction of the main structural members was completed, caused changes in the supporting condition of the mega-truss member and generated the largest stress change in the structural member during construction.

The variations in stress induced by the bent removal were examined through a numerical simulation. A comparison of the measured results produced slightly different results than the quantitative evaluation, but similar tendencies were also observed in the overall stress variations of the members. The accuracy of the analysis can be enhanced by updating the analysis model so that it accurately reflects the measured results, which is one of the main functions of a monitoring system based on sensors.

The comparison of the variations in stress and exterior temperature through temperature sensors indicated that the variations in stress due to temperature were comparable to the changes that resulted from the bent removal. Although temperature stress is temporary, it should be considered a significant factor when extensive variations in temperature occur during construction. The change in the constraint condition of the member, which was caused by the bent removal, was also confirmed to have a large influence on stress change due to temperature variation. The measurement results prove that the WSNS based on VWSGs proposed in this research can be applied as a practical monitoring method to ensure construction safety and the accurate construction of a building.

## Figures and Tables

**Table 1.** Main construction schedule.

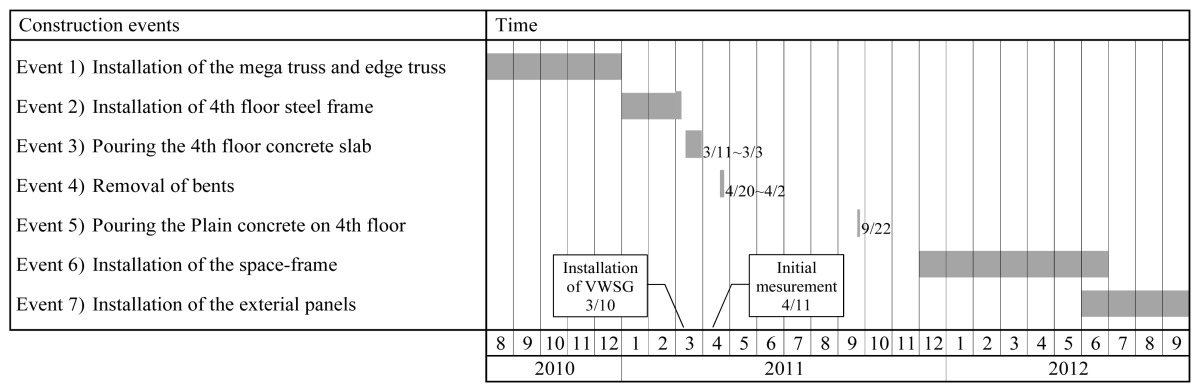

## References

[1] Doherty J.E., Kobayashi A.S. (1987). Nondsestructive Evaluation. Handbook on Experimental Mechanics.

[2] Arditi D., Gunaydin H.M. (1997). Total quality management in the construction process. Int. J. Proj. Manage..

[3] Aktan A.E., Catbas F.N., Grimmelsman K.A., Tsikos C.J. (2000). Issues in infrastructure health monitoring for management. J. Eng. Mech. Asce..

[4] Brownjohn J.M.W. (2007). Structural health monitoring of civil infrastructure. Philos. T. R. Soc. A.

[5] Liu S.C., Tomizuka M. (2003). Vision and Strategy for Sensors and Smart Structures Technology Research.

[6] Fan W., Qiao P. (2011). Vibration-based damage identification methods: A review and comparative study. Struct. Health Monit..

[7] Straser E.G., Kiremidjian A.S. (1998). A Modular, Wireless Damage Monitoring System for Structures.

[8] Lynch J.P., Loh K.J. (2006). A summary review of wireless sensors and sensor networks for structural health monitoring. Shock Vib. Dig..

[9] Krishnamurthy V., Fowler K., Sazonov E. (2008). The effect of time synchronization of wireless sensors on the modal analysis of structures. Smart Mater. Struct..

[10] Qiu Z., Wu J., Yuan S. (2011). A wireless sensor network design and evaluation for large structural strain field monitoring. Meas. Sci. Technol..

[11] Jang S., Sim S.H., Jo H., Spencer B.F. (2012). Full-scale experimental validation of decentralized damage identification using wireless smart sensors. Smart Mater. Struct..

[12] Park H.S., Shin Y., Choi S.W., Kim Y. (2013). An integrative structural health monitoring system for the local/global responses of a large-scale irregular building under construction. Sensors.

[13] DeWolf J.T., Lauzon R.G., Culmo M.P. (2002). Monitoring bridge performance. Struct. Health Monit..

[14] Park K.T., Kim S.H., Park H.S., Lee K.W. (2005). The determination of bridge displacement using measured acceleration. Eng. Struct..

[15] Bao C., Hao H., Li Z. (2013). Vibration-based structural health monitoring of offshore pipelines: Numerical and experimental study. Struct. Control Health Monit..

[16] Kim J.T., Ho D.D., Nguyen K.D., Hong D.S., Shin S.W., Yun C.B., Shinozuka M. (2013). System identification of a cable-stayed bridge using vibration responses measured by a wireless sensor network. Smart Struct. Syst..

[17] Park H.S., Lee H.M., Adeli H., Lee I. (2007). A new approach for health monitoring of structures: Terrestrial laser scanning. Comput. Aided Civ. Inf..

[18] Adewuyi A.P., Wu Z.S., Serker N.H.M.K. (2009). Assessment of vibration-based damage identification methods using displacement and distributed strain measurements. Struct. Health Monit..

[19] Rodrigues C., Felix C., Figueiras J. (2011). Fiber-optic-based displacement transducer to measure bridge deflections. Struct. Health Monit..

[20] Moschas F., Stiros S. (2011). Measurement of the dynamic displacements and of the modal frequencies of a short-span pedestrian bridge using GPS and an accelerometer. Eng. Struct..

[21] Ye X.W., Ni Y.Q., Wai T.T., Wong K.Y., Zhang X.M., Xu F. (2013). A vision-based system for dynamic displacement measurement of long-span bridges: algorithm and verification. Smart Struct. Syst..

[22] Majumder M., Gangopadhyay T.K., Chakraborty A.K., Dasgupta K., Bhattacharya D.K. (2008). Fibre Bragg gratings in structural health monitoring—Present status and applications. Sens. Actuators A: Phys..

[23] Chacón R., Guzmán F., Mirambell E., Real E., Oñate E. (2009). Wireless sensor networks for strain monitoring during steel bridges launching. Struct. Health Monit..

[24] Adewuyi A.P., Wu Z.S. (2010). Modal macro-strain flexibility methods for damage localization in flexural structures using long-gauge FBG sensors. Struct. Control .Health Monit..

[25] Jo H., Park J.W., Spencer B.F., Jung H.J. (2013). Develoment of high-sensitivity wireless strain sensor for structural health monitoring. Smart Struct. Syst..

[26] Kim Y.S., Sung H.J., Kim H.W., Kim J.M. (2011). Monitoring of tension force and load transfer of groundanchor by using optical FBG sensors embedded tendon. Smart Struct. Syst..

[27] Li H.N., Li D.S., Song G.B. (2004). Recent applications of fiber optic sensors to health monitoring in civil engineering. Eng. Struct..

[28] Park H.S., Jung S.M., Lee H.M., Kwon Y.H., Seo J.H. (2007). Analytical models for assessment of the safety of multi-span steel beams based on average strains from long gage optic sensors. Sens. Actuat. A Phys..

[29] Park H.S., Jung H.S., Kwon Y.H., Seo J.H. (2006). Mathematical models for assessment of the safety of steel beams based on average strains from long gage optic sensors. Sens. Actuat. A: Phys..

[30] Deng L., Cai C.S. (2007). Applications of fiber optic sensors in civil engineering. Struct. Eng. Mech..

[31] Pang C., Yu M., Gupta A.K., Bryden K.M. (2013). Investigation of smart multifunctional optical sensor platform and its application in optical sensor networks. Smart Struct. Syst..

[32] Coutts D.R., Wang J., Cai J.G. (2001). Monitoring and analysis of results for two strutted deep excavations using vibrating wire strain gauges. Tunn. Undergr. Sp. Tech..

[33] Yu F., Gupta N. (2005). An efficient model for improving performance of vibrating-wire instruments. Measurement.

[34] Lee H.M., Kim J.M., Sho K., Park H.S. (2010). A wireless vibrating wire sensor node for continuous structural health monitoring. Smart Mater. Struct..

[35] Lee H.M., Park H.S. (2013). Measurement of maximum strain of steel beam structures based on average strains from vibrating wire strain gages. Exp. Tech..

[36] Bourquin F., Joly M. (2005). A magnet-based vibrating wire sensor: Design and simulation. Smart Mater. Struct..

[37] Neild S.A., Williams M.S., McFadden P.D. (2005). Development of a vibrating wire strain gauge for measuring small strains in concrete beams. Strain.

[38] Sreeshylam P., Ravisankar K., Parivallal S., Kesavan K., Sridhar S. (2008). Condition monitoring of prestressed concrete structures using vibrating wire sensors. Int. J. Comadem.

[39] Xia Y., Ni Y.Q., Zhang P., Liao W.Y., Ko J.M. (2011). Stress development of a supertall structure during construction: Field monitoring and numerical analysis. Comput. Aided Civ. Inf..

[40] Choi S.W., Kwon E., Kim Y., Hong K., Par H.S. (2013). A practical data recovery technique for long-term strain monitoring of mega columns during construction. Sensors.

[41] Choi S.W., Kim Y., Kim J.M., Park H.S. (2013). Field monitoring of column shortenings in a high-rise building during construction. Sensors.

[42] Viterbi A.J. (1995). CDMA: Principles of Spread Spectrum Communication.

[43] System, M.U.S.. http://en.midasuser.com/.

